# Brainwave Patterns and Metabolic Adaptations in Rowers Crossing the Atlantic: A Case Series Pilot Study

**DOI:** 10.7759/cureus.74731

**Published:** 2024-11-29

**Authors:** Merin Chandanathil, Daniel P Longman, Tomasz Nowak, Jonathan C.K. Wells, Michael P Muehlenbein, Jay T Stock, Vasavi R Gorantla, Courtney Lewis, Richard M Millis

**Affiliations:** 1 Department of Physiology, American University of Antigua, St. John's, ATG; 2 School of Sport, Exercise and Health Sciences, Loughborough University, Loughborough, GBR; 3 Department of Anthropology, Baylor University, Waco, USA; 4 Childhood Nutrition Research Centre, UCL Institute of Child Health, London, GBR; 5 Department of Anthropology, University of Western Ontario, London, CAN; 6 Department of Medical Education, California University of Science and Medicine, Colton, USA; 7 Department of Clinical Medicine, American University of Antigua, St. John's, ATG

**Keywords:** cortisol, extreme sports, leptin, oxidative stress, quantitative electroencephalography, testosterone, transatlantic rowing

## Abstract

Introduction: This pilot study was designed to test the hypothesis that quantitative electroencephalographic (qEEG) measurements reflect physiological adaptations for brain energy reallocation. The study focused on a team of three well-matched male rowers participating in a 30-day, 2,650-mile continuous transatlantic rowing competition, examining the effects of extreme, prolonged stress on brain function and metabolic adaptations.

Methods: Measurements at the start and finish lines included body weight, height, waist circumference, body fat, and a panel of hormones and biochemical markers. Post-race qEEG parameters were recorded under eyes-open (EO) and eyes-closed (EC) conditions. qEEG data were compared to a reference population (ages 6-90 years) and to an age-matched 27-year-old male medical student serving as a control subject. qEEG analysis evaluated voltage amplitudes, wave distribution patterns, theta-to-beta ratios (TBR), and coherence levels. Hormonal changes and oxidative stress markers were also assessed before and after race.

Results: Two rowers exhibited post-race dominance of high-frequency beta activity, while one displayed co-dominance of delta and beta waves. Compared to the control subject (TBR = 1.25), the rowers’ low TBRs (< 0.2) indicated high vigilance and low relaxation during EC conditions. Cortisol levels increased in all rowers and were associated with beta coherence >1 SD above the reference mean. Testosterone decreased in two rowers but increased in one; the smallest cortisol increase corresponded with the largest testosterone decrement. Decreases in oxidative stress markers correlated with a shift from right- to left-sided alpha asymmetry, consistent with redistribution of alpha wave energy to the nondominant hemisphere. This pattern was also observed in the control subject. Increased testosterone in one rower was linked to a decrease in the percentage of sites exhibiting normal theta frequencies, indicating a potential role for testosterone in brain energy reallocation.

Conclusion: The findings suggest that qEEG measurements reflect physiological adaptations in response to extreme stress, supporting the hypothesis that metabolic energy is reallocated to optimize vigilance and performance. The observed correlations between hormonal changes, oxidative stress markers, and qEEG parameters provide preliminary evidence of mechanisms for brain energy reallocation. These insights highlight the potential for qEEG to identify biomarkers of stress adaptation and lay the groundwork for larger studies to further elucidate these mechanisms.

## Introduction

Ultra-endurance sports impose a significant metabolic challenge [1-5], pushing physiological and cognitive systems to the limits of adaptive plasticity [6]. Such events are engaged in by adventurous individuals, who demonstrate a mindset of facing and embracing fear, with the potential for serious injury, often in remote locations where medical services may be scarce [7,8]. Ocean rowing events, such as those involving crossing the Atlantic Ocean without the aid of motors or sails, demand that participants manage severe negative energy balance arising from prolonged strenuous physical exertion and limited energy intake, sleep deprivation, unpredictable weather conditions, and the psychological strains of geographical isolation [9]. The significant challenges associated with transatlantic rowing challenge the human body at multiple levels, affecting the stress response systems, physiological and mechanical functions, metabolism, appetite, and hormonal balance [10]. Understanding the biological challenges associated with prolonged ultra-endurance events such as transatlantic rowing may represent a valuable opportunity to improve the current understanding of the physiological adaptations by which humans conserve energy during periods of intense and/or prolonged stress. Such work has the potential to contribute to advances in athletic performance, evolutionary anthropology, and broader public health [11-13].

Ultra-endurance sports, as an intense environmental stressor, can significantly alter the electrical and chemical signaling within the brain's stress-sensing systems, such as the hypothalamic-pituitary-adrenal (HPA) axis, by increasing the brain’s fast beta wave activity and modulating cortisol secretion, both of which are associated with energy conservation adaptations during prolonged physical and mental exertion [14]. The human brain consumes approximately 20% of the body's total energy despite representing only about 2% of body mass [15]. During periods of stress, the brain reallocates energy from non-essential processes to those involved in threat detection and response. This reallocation is driven by stress-induced activation of the HPA axis, leading to the release of glucocorticoids such as cortisol [16]. The amygdala, a brain region critical for fear processing, becomes more active under stress, facilitating rapid detection and response to danger [17]. This shift in energy allocation reflects an evolutionary adaptation to prioritize processes aiding survival during acute stress. Vigilance - the ability to detect and respond to potential threats - is one such process that is crucial for survival [18]. By diverting resources towards sensory processing and motor responses, vigilance allows rapid detection of threats such as predators and facilitates evasive action. In the context of the flight-fight stress response, fast beta brainwaves are reported to accompany metabolic oxidative stress where a behavioral state of high vigilance is required [19,20]. Rowers participating in a transatlantic rowing competition presents a model for studying the biological underpinnings of vigilance. Vigilance may represent a behavioral state for optimizing human performance under extreme physical and environmental conditions. The unique environment of transatlantic rowing highlights the interplay between the need for metabolic energy reallocation and the physiological adaptations required to work during prolonged stress [21]. This case series pilot study was, therefore, designed to determine whether the prolonged stress associated with a grueling transatlantic rowing race may be reflected in the rowers’ brainwave signatures. We, therefore, hypothesized that EEG measurements made at the finish line reflect the underlying neurochemical processes, which are influenced by the changes in hormone levels, metabolic energy substrates, and oxidative stress markers [22-25] measured at the start and finish lines of a 30-day row across the Atlantic Ocean.

## Materials and methods

Study purpose and design

This study was approved by the University of Cambridge Human Biology Research Ethics Committee and Institutional Review Board (IRB). The quantitative electroencephalography (qEEG) portion was approved as a pilot study by the American University of Antigua Research Council and the IRB. Participants provided both verbal and written informed consent, using forms approved by the University of Cambridge and the American University of Antigua IRB for scientific validity and ethical considerations. The participants were rowers in the transatlantic racing competition from the starting line at sponsored by the Talisker Atlantic Challenge from the starting line at San Sebastian de La Gomera (Canary Islands) on 14 December 2017 to the finish line at Nelson’s Dockyards in Antigua and Barbuda (Lesser Antilles Islands, West Indies) on 13 January 2018, a total distance of 2,644 nautical miles.

The study was part of the five-year European Research Council-funded ADaPt Project: Adaptation, Dispersals and Phenotype (https://www.arch.cam.ac.uk/research/projects/archived-projects/adapt-project-adaptation-dispersals-and-phenotype). The ADaPt Project is a highly interdisciplinary project seeking to improve current understanding of the origins of human variation by examining the adaptability of living humans to challenging environments. Although this project was based at the University of Cambridge (where both Professor Jay Stock, Principal Investigator, and Dr. Daniel Longman, Post-Doctoral Fellow) were employed at the time, the project’s aims demanded an international collaborative approach. The core project team involved (1) Professor Jonathan Wells (UCL, UK), a long-term collaborator of both Stock and Longman who was closely involved with the ADaPt Project throughout; (2) Professor Richard M. Millis (AUA, Antigua & Barbuda), who provided expertise regarding qEEG as well as essential logistical support at the Project’s field site in Antigua; and (3) Professor Michael Muehlenbein (Baylor University, USA), whose laboratory provided an immune analysis and interpretation of blood samples. The collaboration was managed centrally by the Project Principal Investigator (JS), who held regular meetings with the wider team both online and in person. Sample analysis was delayed by 2+ years due to COVID-19. Two authors (JS and DL) took new jobs, which further delayed the metabolic analyses.

Participants

The demographic, anthropomorphic, and physiological characteristics of the rowing team participants are described separately for each rower, listed as rower #1, rower #2, and rower #3. The group consisted of three well-matched males with similar characteristics. A fourth male member of the team was excluded due to excessive artifacts in his qEEG recordings.

Quantitative electroencephalography (qEEG)

We performed post-race qEEG measurements on a team of three rowers that we identified based on similar characteristics, who shared the same boat, ate the same food, slept, and rowed the same schedules. qEEGs were recorded immediately after 30 days 4 hours 59 minutes of continuous rowing 2,644 nautical miles. We evaluated the qEEG patterns and the plasma levels of three stress-sensitive hormones - cortisol, testosterone, and leptin, sampled within one hour of arriving at the finish line of the Atlantic Challenge at Nelson’s Dockyard, Antigua-Barbuda. qEEG measurements were made while participants were seated upright using a standard electrode cap containing 19 recording electrodes at positions based on the standard 10-20 system attached to a left ear reference electrode clip. qEEG recordings were performed with a computer-based system (Brain Master, Model Discovery 20, Bedford, OH) under eyes-open (EO) and eyes-closed (EC) conditions for five minutes each. Artifacts were removed by visual recognition and with the aid of an online qEEG editing system (New Mind Technologies Inc., Roswell, GA). The qEEG data were based on artifact-free qEEG recordings, which varied from two to three minutes in duration. Magnitudes of qEEG voltage, dominant (mode) frequencies, and inter-hemispheric and intrahemispheric coherences were analyzed in the standard frequency bandwidth ranges of delta (1-3 Hz), theta (4-7 Hz), alpha (8-12 Hz), and beta (13-30 Hz). Theta-to-beta ratios (TBRs) were computed and compared to age-related normal values (normal: 1.5-2.0). TBRs and midline analyses at standard frontal, temporal, parietal, and occipital recording sites were also compared to normal values based on a reference population consisting of 1,000 subjects, 6-90 years of age (New Mind Technologies, Inc., Roswell, GA).

This case series comparing the brainwave patterns of a team of three right-handed male transatlantic rowers (aged 26-29) at the finish line of a 30-day extreme rowing competition necessitated the incorporation of meaningful reference points to draw valid comparisons. We, therefore, employed the typical qEEG data from a broad reference population of healthy males and females aged six to 90 years for comparisons. While this typically provides a general benchmark for what is considered "normal" brain activity for the purpose of neurofeedback training and psychological counseling, it may not capture the nuances of extreme conditions experienced by elite athletes or highly stressed individuals. Therefore, to enhance the relevance of our analysis, we included qEEG data from a 27-year-old right-handed male medical student under academic achievement stress as a reference point. This individual, though not an athlete, shares key demographic similarities with our rowers in terms of age, handedness, and gender. The academic achievement stress experienced by the medical student, while distinct from the physical stress of the rowers, serves as a meaningful substitute for understanding how stress - whether intellectual or physical - might influence brain activity in individuals of similar cognitive and physiological capacity. By comparing the qEEG patterns of the rowers to this non-athlete under academic stress, we sought to explain how extreme physical stressors, like those of transatlantic rowing, affect brain activity differently than intense intellectual demands. This approach provides a more relevant comparison than using a generalized population reference, acknowledging the unique demands faced by our elite athletes and the specialized nature of their physiological and cognitive responses to extreme conditions.

qEEG coherence

qEEG coherences in each bandwidth served as a surrogate measurement of neural interconnectivity. Coherences were computed within the software digital filters for each bandwidth (beta, alpha, theta, delta) as the cross-spectral power normalized to the total spectral power in that band, as a function of time (Brain Master, Model Discovery 20, Bedford, OH). This approach causes the values to range from 0.0 to 1.0, analogous to the Pearson product-moment correlation coefficient (r), but computed in the frequency domain. Decimal fractions were eliminated by expressing each coherence value in r x 100. This coherence is like a correlation coefficient in that it shows the degree of correlation between two signals from symmetrical left and right scalp recording sites. Signals containing identical frequency characteristics exhibit coherence values of 100, and those with different frequency characteristics have coherences < 100, with decreasing values reflecting degrees of difference or dissimilarity. Interhemispheric qEEG coherences were based on the mean coherences recorded from each pair of left- and right-sided prefrontal, frontal-parietal, and occipital recording sites.

qEEG profile

qEEG profiles were constructed for each rower based on quantitative comparisons between each rower’s and the normal reference population’s data. Determination of normality was based on the finding that an individual rower’s mean value was within one standard deviation (1 SD) of the reference population’s mean value. The profile findings for each qEEG parameter were expressed separately for each of the four standard EEG bandwidths (delta 0-3 Hz, theta 4-7 Hz, alpha 8-12 Hz, and beta 13-30 Hz) as follows: (i) mean voltage amplitude was low if found to be more than 1 SD below and high if found to be more than 1 SD above that of the reference population at each of 76 recording sites; (ii) mode (dominant) frequency was slow if more than 1 SD lower and fast if more than 1 SD higher than that of the reference population at each of 76 recording sites; (iii) coherence (interconnectivity) was normal if the coherence (correlation) coefficient for voltage amplitude and phase was within 1 SD, low if more than 1 SD less, and high if more than 1 SD less than that of the reference population at eight paired left- and right-sided prefrontal, frontal, parietal, and occipital recording sites; and (iv) alpha symmetry was even if the mean voltage amplitude was within 1 SD, normal right-sided if the mean voltage amplitude of the right-sided recording site was more than 1 SD larger, and left-sided if the mean voltage amplitude of the left-sided recording site was more than 1 SD larger than that of the reference population, at eight paired left- and right-sided sites.

Biomarkers

The biochemical markers, indicative of various physiological stressors, were measured at the start and finish lines of the race, an interval of approximately 30 days. These markers included the cortisol hormonal HPA axis-generalized stress marker; the leptin hormonal (adipokine) energy balance stress marker; the testosterone sex hormonal stress marker, the collagen oligomeric matrix protein (COMP) musculoskeletal stress marker, and the interleukin-6 (IL-6) proinflammatory cytokine inflammation marker; the myoglobin muscle breakdown marker; the total antioxidant activity marker (TAC) and the malondialdehyde (MDA) oxidative stress marker.

## Results

**Figure 1:**
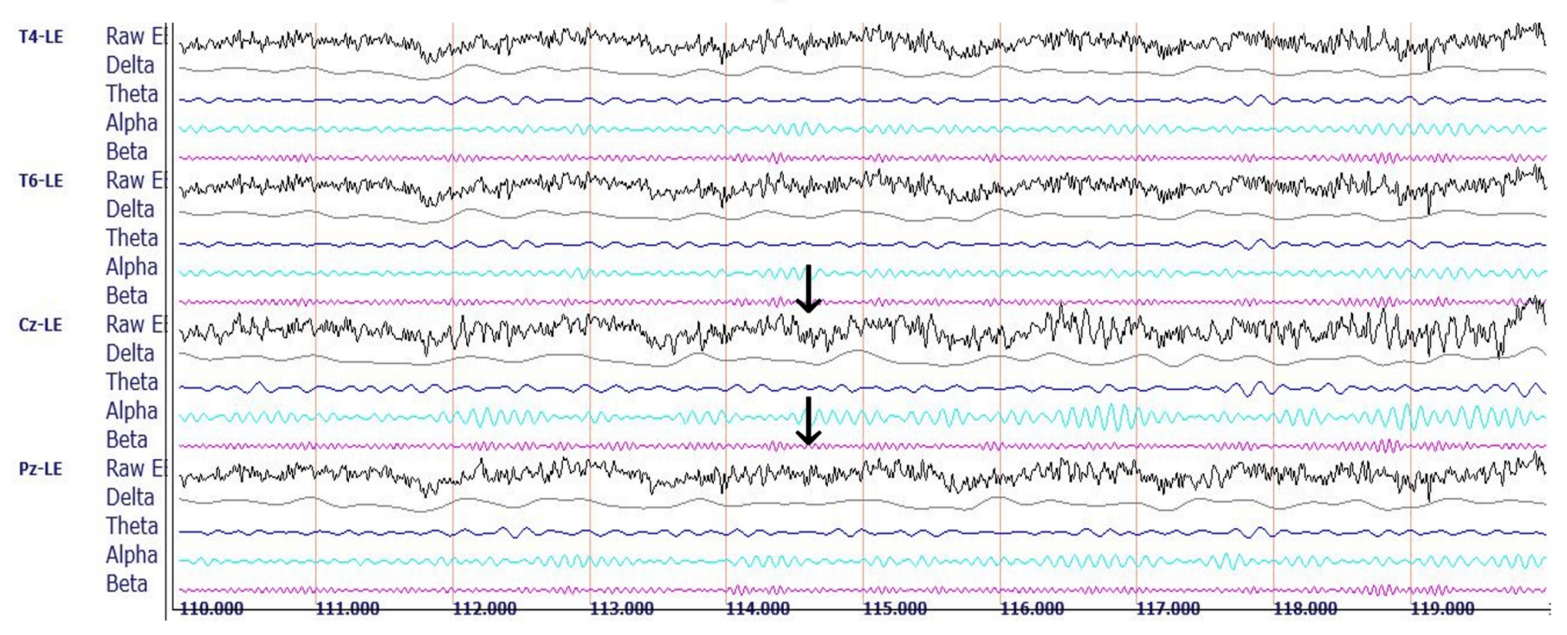
qEEG recording of the control subject. Representative raw EEG and decomposed delta, theta, alpha, and beta brainwave recordings for three of 19 standard recording sites in a 27-year-old male medical student with eyes closed at rest. Sites are based on the international 10-20 system for electrode placement, showing the electrical differences between a reference electrode clipped to the left ear (LE) and electrodes located at the following four sites: T4 (right lateral temporal site overlying the middle temporal gyrus); T6 (right inferior-posterior temporal site overlying the temporal-occipital junction); Cz (central midline site overlying the junction of precentral and postcentral gyri known as the sensorimotor cortex); and Pz (midline electrode posterior to Cz, overlying the parietal cortex). Each horizontal line marks 1s. Arrows point to the central midline channel, demonstrating the raw EEG composite wave recording containing a mixture of all the represented frequencies (top arrow) and the filtered beta wave recording (bottom arrow), useful for a visual semiquantitative comparison between the control subject and the rowers by observing the differences in amplitudes and frequencies.

Figure 1 presents the qEEG recording of the control subject.

**Figure 2:**
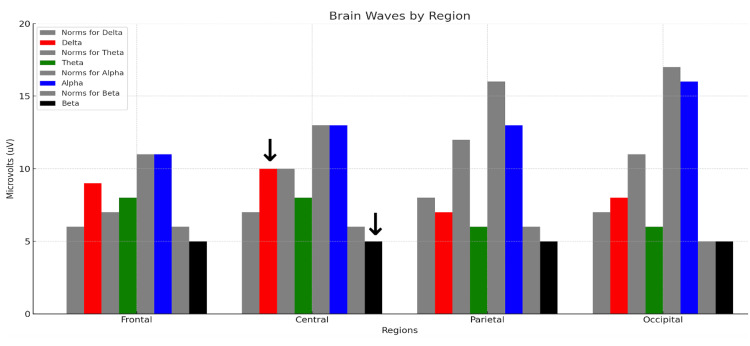
qEEG midline analysis for the control subject. Each colored bar represents the relative voltage amplitude expressed in microvolts (uV) within the standard delta (red, 1-3 Hz), theta (green, 4-7 Hz), alpha (blue, 8-12 Hz), and beta (black, 13-30 Hz) bandwidths recorded from a 27-year-old male medical student with eyes closed at rest. Each neutral grey bar shown to the immediate left of each colored bar represents the normative values for the reference population. The abscissa shows the average qEEG voltage (uV) for each bandwidth computed for the frontal (F3, F4, F7, F8), central midline, parietal midline, and occipital (O1, O2) regions. The arrows over the red bar (delta voltage amplitude) and black bar (beta voltage amplitude) measured at the central midline electrode (Cz) are useful for a visual semiquantitative comparison between the control subject and the rowers.

Figure 2 shows the control subject's midline voltage amplitude analysis.

The control subject’s midline analysis demonstrates variation in the differences between the subject’s voltage amplitudes and those of the reference population at the different recording sites. Using the central (Cz) site as a reference site, the delta, theta, alpha, and beta waves exhibit mean voltage amplitudes relatively close to those of the reference population’s voltages. Comparing all the sites, there is a wide distribution of voltage representative of all the standard frequencies, without a marked dominance of any of the bandwidths. The control subject's average TBR was 1.25 for the EC condition depicted in Figure 2 and 1.25 for the EO condition (not shown).

**Figure 3:**
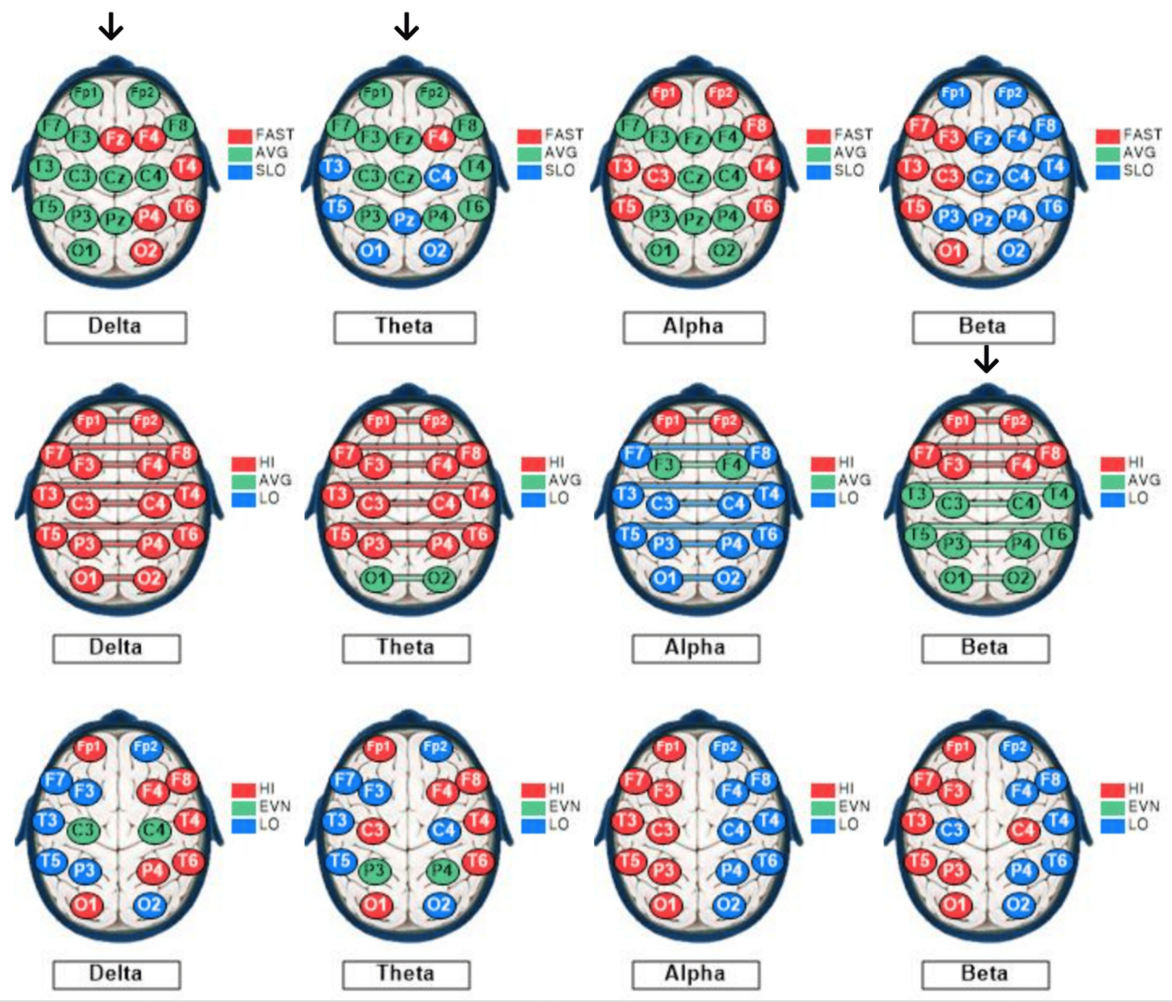
qEEG dominant frequencies, interconnectivities, and asymmetries for the control subject. The qEEG brain maps based on recordings from a 27-year-old male medical student with eyes closed at rest. The maps show sites of variations based on standard deviations (SD) from the means of the reference population for each of the standard EEG bandwidths: delta (1-3 Hz), theta (4-7 Hz), alpha (8-12 Hz), and beta (13-30 Hz). Each EEG recording site is color-coded as follows: green, within 1 SD of the mean; blue more than 1 SD lesser than the mean; and red, more than 1 SD greater than the mean. Top row: Mode (dominant) frequencies. Middle row: Coherences (interconnectivities). Bottom row: Interhemispheric differences in voltage amplitudes (asymmetries). The arrows point to the maps useful for a visual semiquantitative comparison between the control subject and the rowers. Only the delta and theta voltage amplitude and beta-dominant frequency maps exhibit > 50% recording sites within 1 SD of the mean of the reference population, color-coded green.

Figure 3 depicts the control subject's qEEG dominant frequency, interconnectivity, and asymmetry profiles.

The control subject’s qEEG profile included 25% of sites exhibiting slow dominant frequencies in all bandwidths (overall), with 68% of the sites showing slow beta, 32% showing slow theta dominant frequency, and none (0%) showing slow alpha or slow delta dominant frequency, under the EC condition depicted in Figure 3. Alpha dominant frequency was faster than that of the reference population at 42% of the sites. These findings indicate a predominance of slow dominant frequency in the beta (13-30 Hz) bandwidth. The control subject’s overall coherence measurements were high at 59% of the sites under the EC condition depicted in Figure 3. His alpha wave symmetry was right-sided (normal) at 0% and left-sided at 100% of the sites, whereas his beta wave symmetry was left-sided (normal) at 88% and right-sided at 12% of the sites. These findings indicate moderately high interconnectivity between symmetrical left- and right-sided recording sites, with very high left-sided alpha, despite normal left-sided, beta asymmetry.

Table 1 summarizes the pre- vs. post-race percent changes in the anthropomorphic, physiologic, and metabolic markers for each rower.

**Table 1:** Pre- and post-race percent changes in anthropomorphic, physiologic, and metabolic markers. BMI=body mass index; Waist=waist circumference; Fat=Fat mass; TAC=total antioxidant t capacity; MDA=malondialdehyde oxidative stress marker; IL-6=interleukin-6 proinflammatory cytokine inflammation marker; COMP=collagen oligomeric matrix protein musculoskeletal stress marker

	Rower #1	Rower #2	Rower #3
BMI	-10.1%	-9.0%	-9.4%
Waist	-9.2%	-5.1%	-1.2%
Fat	-42.1%	-64.5%	0%
Leptin	-38.5%	+52.6%	Unmeasurable
Testosterone	-57.5%	-15.4%	+26.5%
Cortisol	+44.4%	+109.5%	+162.6%
Myoglobin	+280.8%	-11.5%	+8.2%
TAC	-45.9%	-10.2%	+35.8%
MDA	-35.4%	+8.8%	+14.7%
IL-6	+197.8%	+239.3%	Unmeasurable
COMP	-18.6%	-34.3%	+33.2%

Rower #1

Rower #1 was a 26-year-old man. He was 184.5 cm tall and weighed 98.8 kg. Table 1 shows that his pre- vs. post-race BMI decreased by 10%. He had a 42% decrease in percentage of body fat and a 46% decrease in fat mass. His lean mass increased marginally by 0.36%. Of the three rowers, he had the largest percent decrease in body weight and BMI and was the only one exhibiting no change in percent lean body mass. His plasma leptin decreased by 38.5%. His TAC and MDA oxidative stress markers decreased by 46% and 35%, respectively. He was the team member with the largest decrements in MDA and TAC. His COMP musculoskeletal stress marker decreased by 19.5% and myoglobin increased by 280.8%. His IL-6 inflammatory marker increased by 197.8%. His cortisol increased by 44%, whereas his testosterone decreased by 57.5%. He was the team member exhibiting the largest decrease (-58%) in testosterone. His post-race qEEG showed a threefold elevation in delta voltage and a fivefold elevation in beta voltage magnitude, compared to the normal reference population. He was the team member exhibiting the lowest post-race TBRs (0.07-0.14). He was alert and communicating normally, with no report or indication of cognitive, neurological, or psychological impairment.

**Figure 4:**
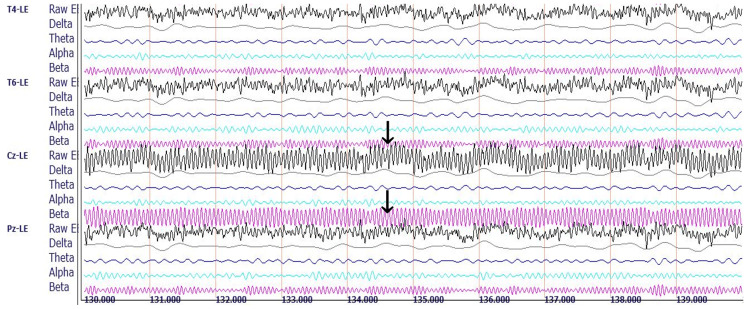
Rower #1's post-race qEEG recording. Representative raw EEG and decomposed delta, theta, alpha, and beta brainwave recordings for three of 19 standard recording sites under eyes-closed conditions. Sites are based on the international 10-20 system for electrode placement, showing the electrical differences between a reference electrode clipped to the left ear (LE) and electrodes located at the following four sites: T4 (right lateral temporal site overlying the middle temporal gyrus); T6 (right inferior-posterior temporal site overlying the temporal-occipital junction); Cz (central midline site overlying the junction of precentral and postcentral gyri known as the sensorimotor cortex); and Pz (midline electrode posterior to Cz, overlying the parietal cortex). Each horizontal line marks 1s. The arrows point to the central midline channel, demonstrating the raw EEG composite wave recording containing a mixture of all the represented frequencies (top arrow) and the filtered beta wave recording (bottom arrow), useful for a visual semiquantitative comparison between Rower #1 and the control subject by observing the differences in amplitudes and frequencies.

Figure 4 shows a representative 10-second qEEG recording from Rower #1.

**Figure 5:**
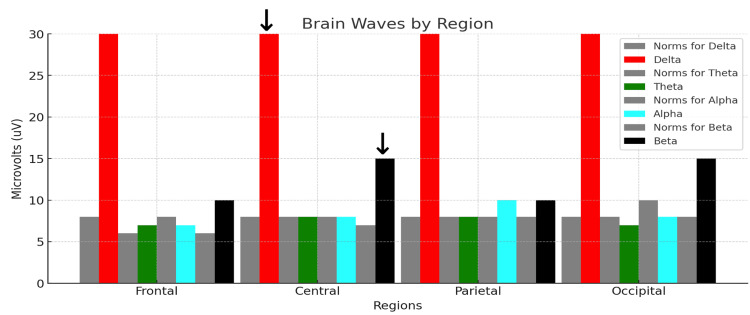
qEEG midline analysis for Rower #1. Each colored bar represents the relative voltage amplitude expressed in microvolts (uV) within the standard delta (red, 1-3 Hz), theta (green, 4-7 Hz), alpha (aqua, 8-12 Hz), and beta (black, 13-30 Hz) bandwidths. Each neutral grey bar shown to the immediate left of each colored bar represents the normative values for the reference population. The abscissa shows the average qEEG data for each bandwidth computed for electrodes in the frontal, central, parietal, and occipital regions. The abscissa shows the average qEEG voltage (uV) for each bandwidth computed for the frontal (F3, F4, F7, F8), central midline, parietal midline, and occipital (O1, O2) regions. The arrows over the red bar (delta voltage amplitude) and black bar (beta voltage amplitude) measured at the central midline electrode (Cz) are useful for a visual semiquantitative comparison between Rower #1 and the control subject.

Figure 5 summarizes Rower #1's post-race midline voltage amplitude analysis.

Rower #1's midline analysis demonstrates the co-dominance of delta and beta waves, with mean voltage amplitudes three- and fivefold larger than those of the reference population at the frontal, temporal, parietal, and occipital sites for the EC condition depicted in Figure 5. TBRs were 0.07-0.14, markedly lower than the reference and control subject's values, for both the EO and the EC conditions.

**Figure 6:**
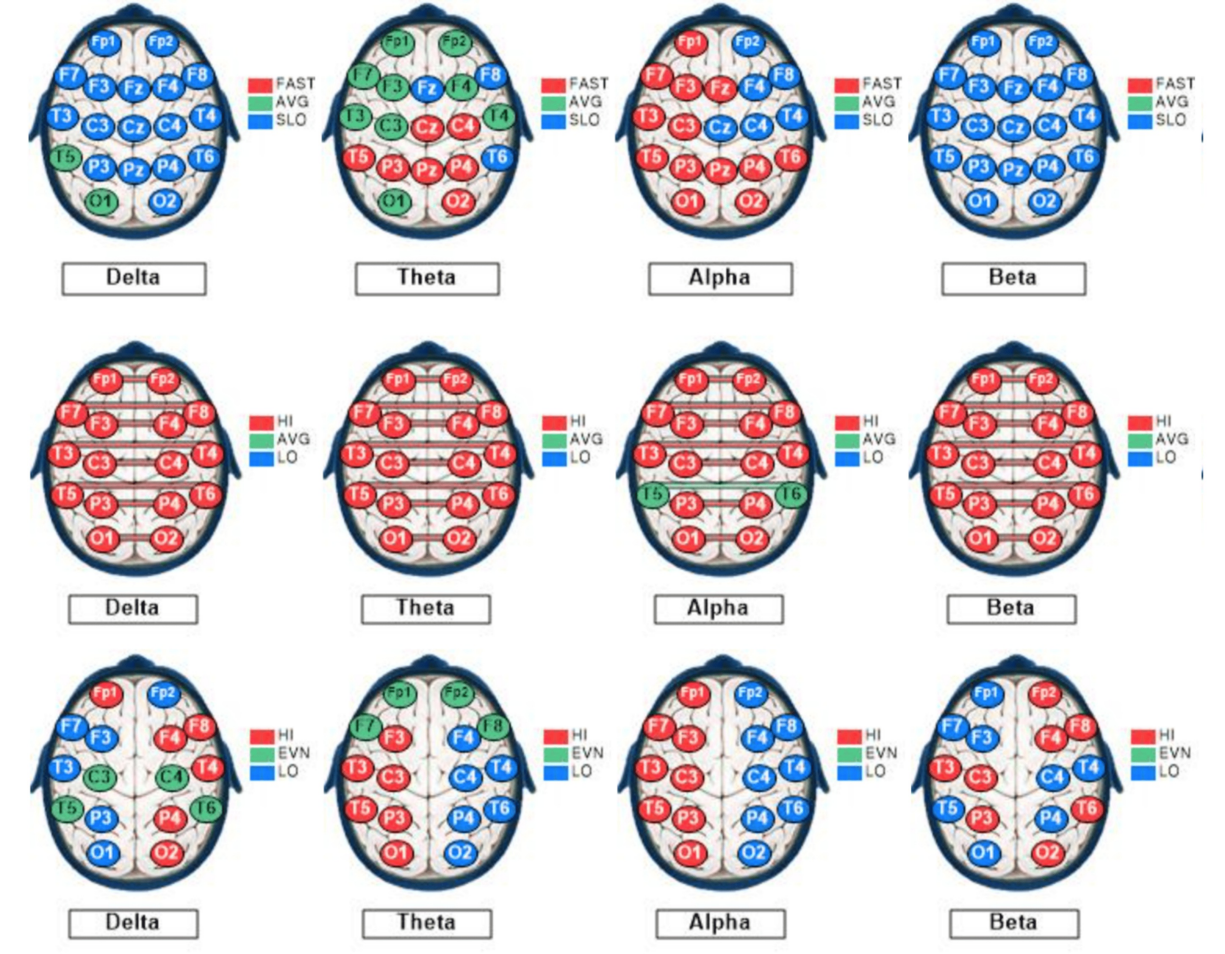
qEEG dominant frequencies, interconnectivities, and asymmetries for Rower #1. The qEEG brain maps show sites of variations based on standard deviations (SD) from the means of the reference population for each of the standard EEG bandwidths: delta (1-3 Hz), theta (4-7 Hz), alpha (8-12 Hz), and beta (13-30 Hz). Each EEG recording site is color-coded as follows: green, within 1 SD of the mean; blue more than 1 SD lesser than the mean; and red, more than 1 SD greater than the mean. Top row: mode (dominant) frequencies. Middle row: Coherences (interconnectivities). Bottom row: Interhemispheric differences in voltage amplitudes (asymmetries). None of Rower #1’s maps show > 50% of recording sites within 1 SD of the mean of the reference population, compared to the control subject with the delta, and theta voltage amplitudes, as well as the beta-dominant frequency map showing > 50% of the recording sites within 1 SD of the reference population, color-coded green.

Figure 6 depicts Rower #1's post-race qEEG dominant frequency, interconnectivity, and asymmetry profiles.

Rower #1’s qEEG profile included 68% of sites exhibiting slow dominant frequencies in all bandwidths (overall), with 100% showing slow beta-dominant frequency and 21% showing slow alpha-dominant frequency under EO conditions. Under EC conditions, the overall dominant frequency was slow at 59% and fast at 26% of the sites. The beta-dominant frequency was slow at all (100%), and alpha-dominant frequency was slow at 32% and was fast at 68% of the sites. These findings indicate a predominance of slow dominant frequencies, especially in the beta (13-30 Hz) bandwidth under both EO and EC conditions, with a shift to predominance of fast alpha-dominant frequency under EC conditions. Rower #1’s overall coherence measurements were high at 97% of the recording sites under both EO and EC conditions. His alpha wave symmetry was right-sided (normal) at 0% and left-sided at 100% of the sites, whereas his beta wave symmetry was normal left-sided at 38% and right-sided at 62% of the sites, under both EO and EC conditions. These findings indicate extremely high interconnectivity between symmetrical left- and right-sided recording sites, a marked reversal of the normal right-sided alpha asymmetry with a moderate reversal of the normal left-sided beta asymmetry.

Rower #2

Rower #2 was a 26-year-old man. He was 186 cm tall and weighed 97.3 kg. Table 1 shows that his pre- vs. post-race BMI decreased by 9%. He had a 64.5% decrease in fat percentage and a 66.5% decrease in fat mass. His lean mass increased by 6.8%, while his dry lean mass decreased by 7.2%. His leptin increased by 52.6%. He was the only team member with an increase (53%) in leptin. His TAC oxidative stress marker decreased by 10%, while MDA increased by 8.8%. His COMP and myoglobin musculoskeletal stress markers decreased by 34% and 11%, respectively. He was the only team member with a decrease (12%) in myoglobin. His IL-6 inflammatory marker increased by 239%. His pre-vs. post-race cortisol increased by 109.5%, whereas his testosterone decreased by 15%. He was one of two team members whose post-race qEEG recording exhibited a marked predominance of beta waves, a sixfold elevation compared to the reference population. His post-race TBRs were 0.17=0.20, markedly lower than those of the reference and control subject's values.

**Figure 7:**
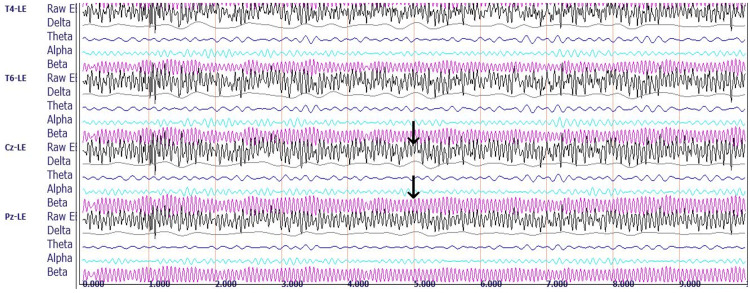
Rower #2's post-race qEEG recording. Representative raw EEG and decomposed delta, theta, alpha, and beta brainwave recordings for three of 19 standard recording sites under eyes-closed conditions. Sites are based on the international 10-20 system for electrode placement, showing the electrical differences between a reference electrode clipped to the left ear (LE) and electrodes located at the following four sites: T4 (right lateral temporal site overlying the middle temporal gyrus); T6 (right inferior-posterior temporal site overlying the temporal-occipital junction); Cz (central midline site overlying the junction of precentral and postcentral gyri known as the sensorimotor cortex); and Pz (midline electrode posterior to Cz, overlying the parietal cortex). Each horizontal line marks 1s. The arrows point to the central midline channel, demonstrating the raw EEG composite wave recording containing a mixture of all the represented frequencies (top arrow) and the filtered beta wave recording (bottom arrow), useful for a visual semiquantitative comparison between Rower #2 and the control subject by observing the differences in amplitudes and frequencies.

Figure 7 shows a representative 10-second qEEG recording from Rower #2.

**Figure 8:**
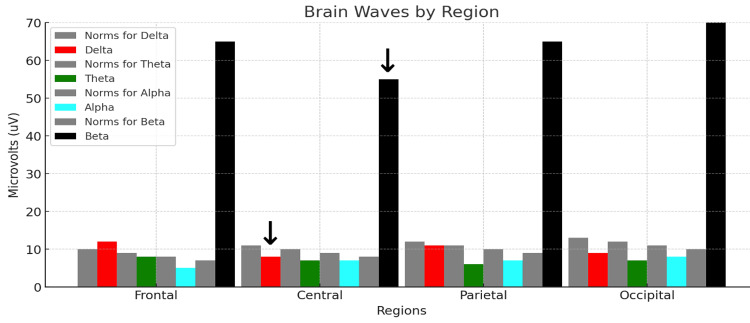
qEEG midline analysis for Rower #2. Each colored bar represents the relative voltage amplitude expressed in microvolts (uV) within the standard delta (red, 1-3 Hz), theta (green, 4-7 Hz), alpha (aqua, 8-12 Hz), and beta (black, 13-30 Hz) bandwidths. Each neutral grey bar shown to the immediate left of each colored bar represents the normative values for the reference population. The abscissa shows the average qEEG data for each bandwidth computed for electrodes in the frontal, central, parietal, and occipital regions. The abscissa shows the average qEEG voltage (uV) for each bandwidth computed for the frontal (F3, F4, F7, F8), central midline, parietal midline, and occipital (O1, O2) regions. The arrows over the red bar (delta voltage amplitude) and black bar (beta voltage amplitude) measured at the central midline electrode (Cz) are useful for a visual semiquantitative comparison between Rower #2 and the control subject.

Figure 8 shows Rower #2's post-race midline voltage amplitude analysis.

Rower #2's midline analysis demonstrates a predominance of beta waves with mean voltage amplitudes sixfold higher than those of the reference population at the frontal, temporal, parietal, and occipital recording sites under the EC condition depicted in Figure 8. TBRs were 0.20 and 0.17, markedly decreased for both the EO and the EC conditions.

**Figure 9:**
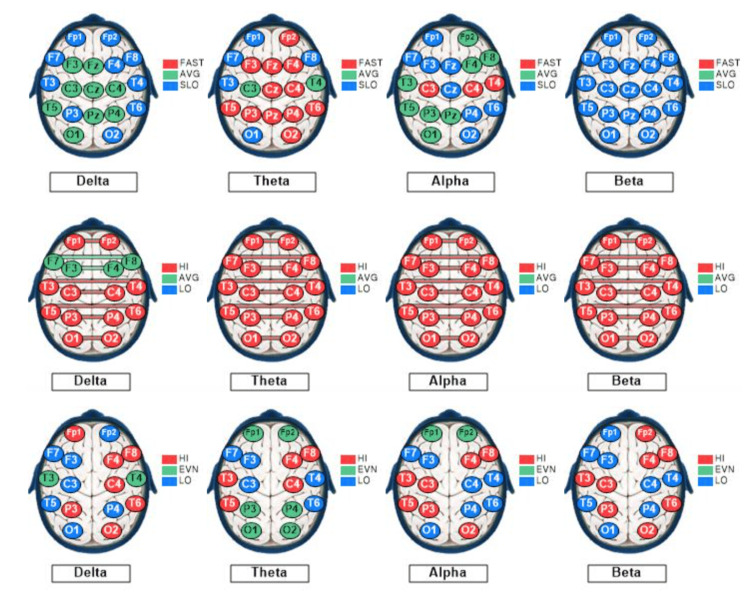
qEEG dominant frequencies, interconnectivities, and asymmetries for Rower #2. The qEEG brain maps show sites of variations based on standard deviations (SD) from the means of the reference population for each of the standard EEG bandwidths: delta (1-3 Hz), theta (4-7 Hz), alpha (8-12 Hz), and beta (13-30 Hz). Each EEG recording site is color-coded as follows: green, within 1 SD of the mean; blue more than 1 SD lesser than the mean; and red, more than 1 SD greater than the mean. Top row: Mode (dominant) frequencies. Middle row: Coherences (interconnectivities). Bottom row: Interhemispheric differences in voltage amplitudes (asymmetries). None of Rower #2’s maps show > 50% of recording sites within 1 SD of the mean of the reference population, compared to the control subject with the delta, and theta voltage amplitudes, as well as the beta dominant frequency map showing > 50% of the recording sites within 1 SD of the reference population, color-coded green.

Figure 9 depicts Rower #2's post-race qEEG dominant frequency, interconnectivity, and asymmetry profiles.

Rower #2’s qEEG profile included 66% of sites exhibiting slow dominant frequencies (overall), with 100% overall showing slow beta-dominant frequency, 21% showing slow alpha-dominant frequency, and 79% showing normal alpha-dominant frequency, under EO conditions. Under EC conditions, the overall dominant frequency was slow at 54% and fast at 21% of the sites. Beta-dominant frequency was slow at all (100%), alpha-dominant frequency was slow at 42%, alpha-dominant frequency was fast at 21%, and alpha-dominant frequency was normal at 25% of the sites. These findings indicate a predominance of slow dominant frequencies, especially in the beta (13-30 Hz) bandwidth, under both EO and EC conditions; however, with a predominance of alpha-dominant frequency found in the normal range under EO conditions, shifting to a predominance of slow alpha-dominant frequency under EC conditions. Rower #2’s overall coherence measurements were high at 94% of the sites under both the EO and the EC conditions. His alpha symmetry was right-sided (normal) at 38% and left-sided at 49% of the sites, whereas his beta symmetry was left-sided (normal) at 38% and right-sided at 49% of the sites, under both the EO and the EC conditions. These findings indicate very high interconnectivity between symmetrical left- and a moderate reversal of normal right-sided alpha and left-sided beta electrical asymmetries.

Rower #3

Rower #3 was a 26-year-old man. He was 177 cm tall and weighed 81.7 kg. His pre- vs. post-race BMI decreased by 9%. There was no change in his fat percentage and a 41% decrease in fat mass. His lean mass decreased by 0.7%. He was the only team member with an increase (+53%) in leptin. His TAC and MDA oxidative stress markers increased by 36%, while his MDA increased by 15%. His COMP and myoglobin increased by 33% and 8%, respectively. His cortisol increased by 163%, and his testosterone increased by 26.5%. He was the team member with the largest increase (+163%) in cortisol. He was one of two team members whose qEEG recording showed a predominance of beta waves, with a five- to sixfold elevation in magnitude, compared to the reference population. His TBR was 0.25, markedly decreased compared to the reference and control subject's values.

**Figure 10:**
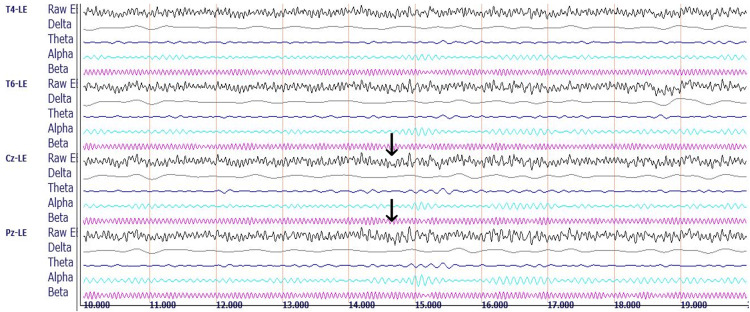
Rower #3's post-race qEEG recording. Representative raw EEG and decomposed delta, theta, alpha, and beta brainwave recordings for three of 19 standard recording sites under eyes-closed conditions. Sites are based on the international 10-20 system for electrode placement, showing the electrical differences between a reference electrode clipped to the left ear (LE) and electrodes located at the following four sites: T4 (right lateral temporal site overlying the middle temporal gyrus); T6 (right inferior-posterior temporal site overlying the temporal-occipital junction); Cz (central midline site overlying the junction of precentral and postcentral gyri known as the sensorimotor cortex); and Pz (midline electrode posterior to Cz, overlying the parietal cortex). Each horizontal line marks 1s. The arrows point to the central midline channel, demonstrating the raw EEG composite wave recording containing a mixture of all the represented frequencies (top arrow) and the filtered beta wave recording (bottom arrow), useful for a visual semiquantitative comparison between Rower #3 and the control subject by observing the differences in amplitudes and frequencies.

Figure 10 shows a representative 10-second qEEG recording from Rower #3.

**Figure 11:**
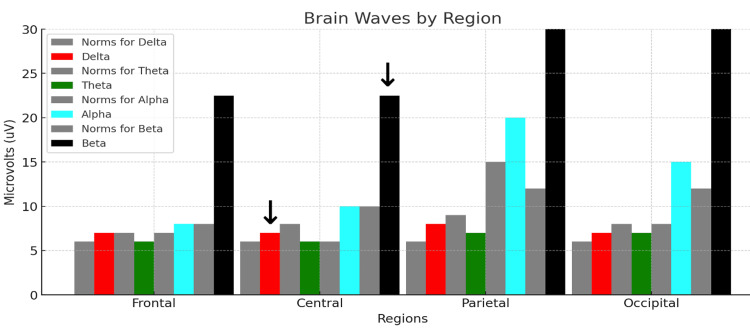
qEEG midline analysis for Rower #3. Each colored bar represents the relative voltage amplitude expressed in microvolts (uV) within the standard delta (red, 1-3 Hz), theta (green, 4-7 Hz), alpha (aqua, 8-12 Hz), and beta (black, 13-30 Hz) bandwidths. Each neutral grey bar shown to the immediate left of each colored bar represents the normative values for the reference population. The abscissa shows the average qEEG data for each bandwidth computed for electrodes in the frontal, central, parietal, and occipital regions. The abscissa shows the average qEEG voltage (uV) for each bandwidth computed for the frontal (F3, F4, F7, F8), central midline, parietal midline, and occipital (O1, O2) regions. The arrows over the red bar (delta voltage amplitude) and black bar (beta voltage amplitude) measured at the central midline electrode (Cz) are useful for a visual semiquantitative comparison between Rower #3 and the control subject.

Figure 11 summarizes Rower #3's post-race qEEG midline voltage amplitude analysis.

Rower #3's midline analysis demonstrates a predominance of beta waves with mean voltage amplitudes five to sixfold higher than those of the reference population at the frontal, temporal, parietal, and occipital recording sites. The TBR was 0.25, markedly decreased for both EO and EC conditions.

**Figure 12:**
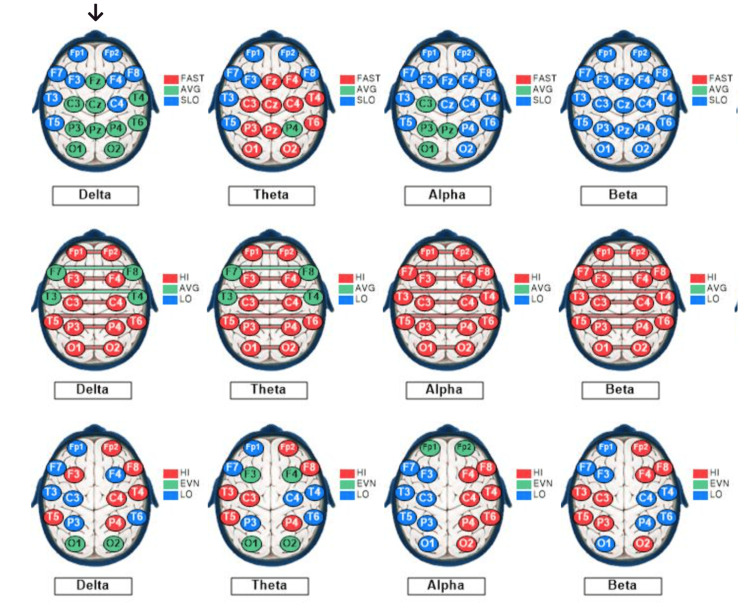
qEEG dominant frequencies, interconnectivities, and asymmetries for Rower #3. The qEEG brain maps show sites of variations based on standard deviations (SD) from the means of the reference population for each of the standard EEG bandwidths: delta (1-3 Hz), theta (4-7 Hz), alpha (8-12 Hz), and beta (13-30 Hz). Each EEG recording site is color-coded as follows: green, within 1 SD of the mean; blue more than 1 SD lesser than the mean; and red, more than 1 SD greater than the mean. Top row: Mode (dominant) frequencies. Middle row: Coherences (interconnectivities). Bottom row: Interhemispheric differences in voltage amplitudes (asymmetries). The arrow points to the delta voltage amplitude map useful for a visual semiquantitative comparison between Rower #3 and the control subject. Only the delta voltage amplitude map shows > 50% of its recording sites within 1 SD of the mean of the reference population, compared to the control subject with the delta, and theta voltage amplitudes, as well as the beta dominant frequency map showing > 50% of the recording sites within 1 SD of the reference population, color-coded green.

Figure 12 depicts Rower #3's post-race qEEG dominant frequency, interconnectivity, and asymmetry profiles.

Rower #3’s qEEG profile included 84% of sites exhibiting slow dominant frequencies (overall), with 100% showing slow beta-dominant frequency and 100% showing slow alpha-dominant frequency under EO conditions. Under EC conditions, the overall dominant frequency was found to be slow at 66% and fast at 14% of the recording sites. Alpha- and beta-dominant frequencies were slow at all (100%) sites. These findings indicate a predominance of slow dominant frequencies, in both the alpha (4-7 Hz) and the beta (13-30 Hz) bandwidths, under both the EO and the EC conditions. Rower #3’s overall coherence measurements were high at 75% and 87% of the sites under EO and EC conditions, respectively. His alpha wave symmetry was right-sided (normal) at 88% and 91% left-sided under EO and EC conditions, respectively. His beta wave symmetry was left-sided (normal) at 50% and right-sided at 50% of the sites, under both the EO and the EC conditions. These findings indicate very high interconnectivity between symmetrical left and a moderate reversal of the normal right-sided alpha and left-sided beta asymmetries.

## Discussion

Pyramidal neurons of the cerebral cortex appear to be the main source of brainwaves [26]. When a million or so pyramidal neurons fire at the same time, the resulting synchronization produces action potentials that summate, thereby resulting in brainwaves [27]. The degree of synchronization influences the strength (voltage amplitude, measured in uV) of each brainwave [28]. Brainwaves are categorized into different frequencies based on the interactions of the action potentials arising from multiple neuronal circuits involving both excitatory pyramidal glutamatergic neurons and inhibitory GABAergic interneurons [29]. Brainwaves change in frequency and amplitude in response to different cognitive tasks, sensory inputs, and levels of arousal [30,31]. For example, changes in attention or sleep states produce shifts in the patterns of brainwave frequencies (expressed in Hz) and voltage amplitudes (expressed in mV). From gamma to delta, the categories are based on the following criteria: delta (1-3 Hz, 20-200 uV), theta (4-7 Hz, 10-100 uV), alpha (8-13 Hz, 20-60 uV), beta (13-30 Hz, 5-30 uV), and gamma (30 Hz and above, 5-25 uV). We did not consider the changes in gamma reliable for this study because of their known interferences with skeletal muscle activity [32]. The slowest brainwaves are delta and theta. Normal delta waves are typically associated with deep sleep, whereas normal theta waves are common in sleep and quiet focus, including declarative memory tasks [33,34]. Normal alpha waves are linked with states of relaxation [35,36]. The fastest brainwaves are beta and gamma. Normal beta waves are associated with active thinking, problem-solving, etc., whereas normal gamma waves are involved in higher mental activity and integration of complex thoughts [30,37]. Brainwave amplitude, frequency, and brain energy demand are thought to be proportional to cognitive requirements [38]. This case series pilot study was designed to determine whether qEEG measures of brainwave voltage and frequency would be indicative of each participant's cognitive requirement for vigilance and reallocation of metabolic energy associated with the intense stress of a 30-day continuous transatlantic rowing competition.

TBR and vigilance

The rowers’ TBR were all <0.2. This is indicative of high vigilance when measured with eyes closed during a state of rest-wakefulness [39] and is lower than that observed in the control subject (1.25) and the normal range of the reference population (>1.0).

Beta coherence and energy conservation

The synchrony between beta waves across right-left symmetrical recording sites (coherence) is shown to be a reliable measure of cognitive processes requiring vigilance [40]. In the present case series, the control subject's beta coherences were within one standard deviation of the reference population's coherences in 5/8 (63%) and more than one standard deviation higher at 3/8 (37%) of his qEEG recording sites. Neural synchronization, as indexed by the qEEG measure of coherence, is thought to be an indicator of neural interconnectivity and a mechanism to improve the efficiency of neural communication [41]. Because synchronous firing requires less metabolic energy than asynchronous neural activity [42], increased coherence should reduce the energy cost of transferring information between cerebral hemispheres. Energy conservation is essential in a stressful environment such as that encountered in a 30-day transatlantic rowing competition, wherein energy demands are high. High beta coherence under stress may, therefore, represent a shift toward more energy-efficient cognitive information processing, allowing the brain to maintain functionality while minimizing metabolic costs. This aligns with the concept of "neural efficiency," where individuals with high coherence and synchronization patterns may use less energy to achieve cognitive goals [43]. The rowers’ cortisol increases ranged from +44% to +162%. These large increases in cortisol were associated with beta coherences > 1 SD above the means of the control subject and of the reference population values in all three rowers. These observations suggest a physiological association between cortisol and beta coherence that is consistent with the "allostatic load" model, where the brain adapts to stress by reallocating resources to maintain functionality under conditions of high stress [44].

Theta-dominant frequency, testosterone, and the energy conservation hypothesis

Theta voltage amplitude increases during prolonged expiration, associated with increased parasympathetic output from the brain, which can create a state of relaxation [45]. Our control subject exhibited normal theta-dominant frequency at 12/19 (63%) of his qEEG recording sites, more than 1 SD slower than normal at 6/19 (32%), and more than 1 SD faster than normal at 1/19 (5%) of sites. In contrast, Rower #1's theta-dominant frequency was normal at 9/19 (47%), more than 1 SD slower than the reference population's at 3/19 (16%), and more than 1 SD faster than the reference population's at 7/19 (37%) of sites. Among the three rowers, Rower #1's qEEG changes were associated with the largest pre- vs. post-race decrement (-57%) in testosterone. Rower #2 had a moderate decrement (-15%) in testosterone, associated with theta-dominant frequencies at qEEG recording sites that were 2/19 (11%) normal, 5/19 (26%) > 1 SD slower, and 12/19 (63%) > 1 SD faster than those of the reference population. Rower #3's theta-dominant frequency profile was markedly different, associated with a (+25%) increase in testosterone; his theta-dominant frequencies were found to be average at 1/19 (5%), slower at 7/19 (37%), and faster at 11/19 (58%) of sites, compared to the reference population. These observations suggest an association of higher testosterone with a lower percentage of sites having normal theta-dominant frequency, thereby shifting to either faster or slower theta-dominant frequencies for cognitive tasks.

The shifts to faster or slower theta-dominant frequencies are likely linked to compensations for theta voltage amplitude changes in the opposite direction, that is, a slower dominant frequency associated with larger voltage amplitude, and vice versa. Such modulations would maintain a constant, homeostatic energy balance for neural signaling [46]. Declarative memory retrieval is one of the known functions of the brain’s theta oscillations [47], indispensable for transatlantic rowers facing prolonged, high-stress competition. Their ability to recall navigational details, tactical adjustments, emergency procedures, and motivational cues can significantly affect their performance and safety. While extreme stress can impair this cognitive process, well-prepared rowers are likely to need an excellent memory as a tool for optimizing their actions, decision-making, and overall endurance. The role of testosterone in modulating the brain’s theta activity has been studied in both experimental animals and humans. Testosterone administration is reported to increase theta voltage amplitude and lower theta-dominant frequency under stress [48]. Under the extreme stress of transatlantic rowing, the brain may prioritize survival by minimizing energy expenditure in neural circuits, particularly those involved in higher-order cognitive processing. Modulation of theta rhythms by testosterone may contribute to this shift because theta oscillations are less energetically demanding than faster oscillations such as beta or gamma [46]. This adaptation could be particularly relevant in survival contexts, where conservation of energy is critical. The shift to theta-dominant rhythms, facilitated by testosterone, could be an evolutionary advantage, allowing individuals under stress to maintain essential cognitive functions - such as attention, memory, and decision-making - while reducing overall metabolic costs. This energy conservation mechanism could be important in long-term stress situations, where maintaining high levels of cognitive processing would otherwise lead to exhaustion or cognitive burnout. Such a neurobiological adaptation likely serves to maintain cognitive function while minimizing metabolic demands, enabling individuals to cope more effectively with prolonged or intense stress. These preliminary findings in transatlantic rowers suggest a line of future research focusing on the interplay between testosterone, stress, and brainwave dynamics to uncover new insights into the hormonal regulation of brain energy efficiency.

Alpha asymmetry and oxidative stress

The pre- vs. post-race changes in the oxidative stress markers MDA and TAC were associated with the post-race percentage of sites exhibiting normal right-sided alpha asymmetry. Rower #1's marked reversal of the normal right-sided alpha asymmetry to 100% left-sided alpha asymmetry was associated with 35%-46% decrements in MDA and TAC, whereas Rower #3's 88% normal right-sided alpha asymmetry was associated with 15%-36% increments in MDA and TAC. Rower #2's associations between these oxidative stress markers and alpha asymmetry were intermediate to those of Rowers #1 and #3. EC alpha frequencies are thought to reflect the activity of the brain’s default mode network (DMN), predominantly active when a person is relaxed and not engaged in complex cognitive, communication, or problem-solving tasks [49]. Electrical (qEEG) symmetry-asymmetry is based on right-left interhemispheric differences in voltage amplitude. Because the DMN is strongly affected by information from the visual pathways and cortex, the amplitude of alpha waves is inversely related to cortical activation. Thus, normal right-sided alpha qEEG asymmetry is indicative of higher alpha voltage amplitude on the right than on the left and, consequently, indicative of greater left-sided cortical activation [50]. This interpretation is consistent with what we know about left cortical dominance associated with right-handedness in the majority of humans, and in most left-handed ones as well, wherein the larger planes of the left cerebral hemisphere's language centers determine dominance for cognitive functions [51]. Hence, the finding of 100% left-sided alpha asymmetry in Rower #1, associated with relatively large decrements in the oxidative stress markers, suggests greater cortical activation of the right hemisphere at rest and that this shift might be correlated with oxidative stress. These findings appear to support the hypothesis that qEEG measures of cerebral cortical functions may be associated with energy conservation adaptations during the extreme stress of transatlantic rowing. The energy conservation hypothesis is also supported by the findings of slower alpha-dominant frequencies, compared to the control subject and the reference population, in all the participant rowers studied with eyes closed during wakefulness. The accumulation of reactive oxygen species (ROS) during oxidative stress is shown to be a stimulus for the rejuvenating and repair functions of the brain's "glymph" system, activated mainly during sleep. In preparation for sleep, the voltage amplitudes of alpha and theta brainwaves are reported to be larger in the right than in the left hemisphere associated with a state of calm wakefulness and relaxation [52,53]. This linkage of the right cerebral cortex is also shown to reflect the predominant role of the right cerebral hemisphere in autonomic and emotional regulation. For example, the right cerebral hemisphere appears to be more involved in activating the parasympathetic branch of the autonomic nervous system (PNS) for "rest and digest" functions [54]. The PNS, linked to right hemispheric activation and alpha asymmetry, appears therefore to promote the conservation of energy, to reduce stress responses, and to induce relaxation, preparing the body for sleep [53]. This interpretation is consistent with the potential for an energy reallocation adaptation related to oxidative stress in the rowers.

Delta voltage amplitude, cortisol, and circadian rhythms

Rower 1’s pre- vs. post-race plasma cortisol was the largest increase. Cortisol is a key marker for the physiological stress associated with changes in circadian rhythms, controlled largely by suprachiasmatic nucleus (SCN) regulation of the pineal gland’s secretion of melatonin [55]. In that regard, there is an important relationship between cortisol-related neuroendocrine signaling and environmental light-dark cycling. Disruptions in the HPA regulatory axis for cortisol caused by stress, irregular sleep patterns, or excessive light exposure are known to be factors in sleep disorders, mood disturbances, and metabolic dysregulation [56]. It is, therefore, plausible that prolonged exposure to visible blue light and ultraviolet (UV) radiation, particularly during an intense and sustained activity such as transatlantic rowing for a month or more can have significant physiological and enduring neurological impacts. This hypothesis is supported by previous studies demonstrating how light exposure affects photosensitive ganglion cells, circadian rhythms, and brainwave activity [57]. Photosensitive retinal ganglion cells (pRGCs) contain melanopsin, a photopigment sensitive to blue light (wavelengths around 480 nm). These pRGCs regulate circadian rhythms through their influence on the SCN associated with the hypothalamus. The SCN is the primary circadian pacemaker in mammals, regulating the sleep-wake cycle and other daily physiological rhythms. Excessive exposure to blue light, especially at night, suppresses melatonin secretion, often leading to circadian rhythm disruptions involving alteration of sleep patterns and the development of sleep disorders [58,59]. The primary focus of prior research has been on the structural damage caused by UV radiation, such as cataracts and macular degeneration. However, there is evidence that UV can also affect retinal cells, including the pRGCs. We speculate that in the rower exhibiting markedly elevated delta activity, prolonged exposure to blue light and UV radiation may have led to super-synchronized brainwaves manifested by a predominance of delta waves during wakefulness [60]. Delta waves are typically associated with deep sleep and are characterized by high voltage amplitude and low frequency. However, sleep deprivation and irregular sleep patterns are reported to increase delta wave activity during wakefulness, a phenomenon known as "sleep intrusions" suggesting that delta activity may function as a “sleep need” gauge [61]. Persistent increases in delta activity during wakefulness also appear to be a feature of traumatic brain injuries [62]. Increased delta activity during wakefulness is also a prominent feature in the EEGs of retired professional American football players impacted by neuropsychological deficits [63] and in kickboxers [64].

There is evidence that excessive blue light may decrease the voltage amplitudes of delta, theta, and alpha waves and increase those of beta waves, depending on the time course of the exposure [65]. In the present study, Rowers #2 and #3 exhibited a predominance of beta activity, and it is noteworthy that they also had relatively large pre- vs. post-race increases in cortisol. Beta waves are typically associated with active thinking, focus, and states of anxiety or extreme vigilance. Previous studies have shown that blue light exposure, especially during nighttime, can disrupt sleep and circadian rhythms, leading to increased alertness and potentially causing anxiety and stress [58]. Such a heightened state of alertness is often associated with increased beta wave activity [66]. The TBR is a metric commonly used in neurofeedback and brainwave studies to assess the balance between relaxation (theta) and alertness (beta) [67], and there is some evidence that prolonged exposure to blue light can cause an imbalance favoring faster frequencies such as beta waves over slower ones such as theta waves [68].

The present case series pilot study of three transatlantic rowers sharing the same boat is an extension of our case series based on five male firefighters living under the same conditions and sharing the same work and call schedule during a 72-hour shift [69]. Both studies highlight the complex interplay between cortisol and testosterone in males subjected to extreme environmental stressors. The firefighters' case series focused on diurnal cortisol and testosterone adaptations in response to the unique challenges of sleep deprivation and exposure to traumatic events during 72-hour work shifts. The present rowers' case series extends this investigation to the prolonged and intense physical and psychological stressors experienced during a 30-day transatlantic rowing competition. In both studies, the patterns of cortisol and testosterone responses underscore the body's attempt to adapt to continuous stress. The firefighters exhibited varied cortisol awakening responses (CAR) with adaptations reflecting either physiological resilience or maladaptation, as evidenced by blunted or exaggerated responses. Similarly, the rowers' case series reveals how extended physical exertion under extreme conditions impacts cortisol regulation, potentially as a mechanism for energy conservation and stress management. These case series support our ongoing interest in elucidating how the interactions between cortisol and testosterone may serve as biomarkers for stress resilience or vulnerability, in individuals exposed to prolonged, extreme environmental stressors. The findings contribute to our understanding of stress-related health risks and adaptations, with implications for interventions aimed at improving physiological resilience in high-stress occupations and activities.

Limitations

These observations are based on a small sample and are subject to the inherent limitations of case series research. For example, the small number of three allowed only for observations of associations between qEEG parameters and biomarkers, but did not permit meaningful quantification of correlation. The study design allowed for only post-race qEEG measurements, which limited our ability to assess pre-existing brain conditions. Consequently, we cannot rule out the possibility that the observed midline qEEG abnormalities, characterized by voltage magnitudes outside the range of normal distributions, may be attributable to prior brain injuries. Additionally, the absence of information regarding participants' dietary supplementation, drug prescriptions, alcohol or other substance ingestion, and post-race psychological evaluation represents a limitation of the present pilot study. These factors could potentially influence qEEG parameters and biomarkers and should be carefully considered in the design of future investigations. Despite these limitations, our findings provide observations that certain biomarkers may serve as potential indicators of brain function alterations associated with extreme stress. These insights underscore the need for future, larger studies to validate and expand upon these observations.

## Conclusions

This case series pilot study is the first to report physiological changes within the members of an elite transatlantic rowing team of three men of similar age and training, the same gender, sharing the same boat environment, diet, and sleep-wake and rowing work schedule. The patterns observed highlight the value of using transatlantic rowers as an experimental model to study the interactions between brainwaves, stress hormones, and markers of oxidative stress. Future research, employing a larger sample size of ocean rowers and taking measurements both pre- and post-race, are required to further elucidate these interactions. In this case series, we observed that post-race predominance of beta waves was associated with high interhemispheric beta coherences, very low TBRs, and pre- vs. post-race increases in cortisol. These findings suggest an impact of stress on brainwaves associated with alertness mediated by the HPA axis. Pre- vs. post-race changes in testosterone were associated with shifts away from a normal percentage of qEEG recording sites exhibiting normal reference ranges of theta dominant frequencies. An association between oxidative stress biomarkers and left-sided alpha asymmetry in one rower indicated a shift of alpha activity to the non-dominant right hemisphere. Because of the limitations of this small case series, it was beyond its scope to determine whether there were significant correlations between the post-race qEEG variables and either the post-race or pre- vs. post-race changes in biomarker levels. Such correlations should be in the scope of a future study to help identify the most likely energy reallocation mechanisms involved in transatlantic rowing. However, the case series offers novel insights into adaptations to the stresses associated with human performance during the ultra-endurance sport of transoceanic rowing. Such adaptations may be relevant to understanding the physiological responses to other extreme sports and environmental stressors.
